# Clinical and Imaging Characteristics of Parkinson's Disease with Negative Alpha‐Synuclein Seed Amplification Assay

**DOI:** 10.1002/mds.70197

**Published:** 2026-01-28

**Authors:** Sarah M. Brooker, Jacopo Pasquini, Seung Ho Choi, David‐Erick Lafontant, Seyed‐Mohammad Fereshtehnejad, Yashar Zeighami, Piergiorgio Grillo, Giulietta M. Riboldi, Houman Azizi, Roqaie Moqadam, Un Jung Kang, Kelly N.H. Nudelman, Andrew Siderowf, Caroline M. Tanner, Thomas F. Tropea, Tatiana Foroud, Lana M. Chahine, Brit Mollenhauer, Kalpana M. Merchant, Douglas Galasko, Christopher S. Coffey, Roseanne D. Dobkin, Ethan G. Brown, Roy N. Alcalay, Daniel Weintraub, Kenneth Marek, Tanya Simuni, Paulina Gonzalez‐Latapi, Nicola Pavese, Kathleen L. Poston, the Parkinson's Progression Markers Initiative

**Affiliations:** ^1^ Department of Neurology Northwestern University Feinberg School of Medicine Chicago Illinois USA; ^2^ Clinical Ageing Research Unit Newcastle University Newcastle upon Tyne UK; ^3^ Department of Clinical and Experimental Medicine University of Pisa Pisa Italy; ^4^ Department of Biostatistics, College of Public Health University of Iowa Iowa City Iowa USA; ^5^ Division of Neurology Toronto Western Hospital, University of Toronto Toronto Ontario Canada; ^6^ Edmond J. Safra Program in Parkinson's Disease and the Morton and Gloria Shulman Movement Disorders Clinic Toronto Western Hospital, UHN Toronto Ontario Canada; ^7^ Department of Neurobiology, Care Sciences and Society (NVS) Karolinska Institute Stockholm Sweden; ^8^ Douglas Mental Health University Institute, Department of Psychiatry McGill University Montreal Quebec Canada; ^9^ Integrated Program in Neuroscience McGill University Montreal Quebec Canada; ^10^ The Marlene and Paolo Fresco Institute for Parkinson's and Movement Disorders Department of Neurology, NYU Langone Health New York New York USA; ^11^ Department of Brain and Behavioral Sciences University of Pavia Pavia Italy; ^12^ IRCCS Mondino Foundation Pavia Italy; ^13^ Department of Neurology NYU Grossman School of Medicine New York New York USA; ^14^ Neuro (Montreal Neurological Institute‐Hospital) McGill University Montreal Quebec Canada; ^15^ Department of Neuroscience NYU Grossman School of Medicine New York New York USA; ^16^ Parekh Center for interdisciplinary Neurology NYU Langone Health New York New York USA; ^17^ Department of Medical and Molecular Genetics Indiana University School of Medicine Indianapolis Indiana USA; ^18^ Department of Neurology University of Pennsylvania Philadelphia Pennsylvania USA; ^19^ Department of Neurology Weill Institute for Neurosciences, University of California San Francisco San Francisco California USA; ^20^ Institute for Neurodegenerative Disorders New Haven Connecticut USA; ^21^ Department of Neurology University of Pittsburgh Pittsburgh Pennsylvania USA; ^22^ Department of Neurology University Medical Center Göttingen Göttingen Germany; ^23^ Paracelsus‐Elena‐Klinik Kassel Kassel Germany; ^24^ Department of Neurosciences University of California San Diego La Jolla California USA; ^25^ Department of Psychiatry Rutgers University, Robert Wood Johnson Medical School Piscataway New Jersey USA; ^26^ Division of Movement Disorders, Department of Neurology Tel Aviv Soursaky Medical Center Tel Aviv Israel; ^27^ Columbia University Irving Medical Center New York New York USA; ^28^ Department of Psychiatry University of Pennsylvania School of Medicine Philadelphia Pennsylvania USA; ^29^ Philadelphia Department of Veterans Affairs Parkinson's Disease Research, Education and Clinical Center Philadelphia Pennsylvania USA; ^30^ Department of Neurology and Neurological Sciences Stanford University Stanford California USA; ^31^ Department of Neurosurgery Stanford University Stanford California USA; ^32^ Wu Tsai Neuroscience Institute Stanford University Stanford California USA; ^33^ Knight Initiative for Brain Resilience Stanford University Stanford California USA

**Keywords:** Parkinson's disease, alpha‐synuclein, biomarkers, parkinsonism, neuroimaging

## Abstract

**Background:**

The cerebrospinal fluid alpha‐synuclein seed amplification assay (CSFasynSAA) detects alpha‐synuclein aggregation in over 90% of individuals with sporadic PD (sPD). However, the clinical characteristics of sPD with negative CSFasynSAA remain undefined.

**Objectives:**

Describe clinical and neuroimaging characteristics of CSFasynSAA‐negative sPD individuals in the Parkinson's Progression Markers Initiative (PPMI).

**Methods:**

We identified sPD PPMI participants with a negative CSFasynSAA (SAA−, n = 80) or positive CSFasynSAA (SAA+, n = 856) result at baseline. For comparative analysis between groups, we used a reduced dataset (n = 79 SAA− and n = 237 SAA+) propensity‐score matched on age, sex, and time since clinical diagnosis. Clinical parameters, dopamine transporter‐single photon emission computed tomography (DAT‐SPECT), and magnetic resonance imaging (MRI) brain volumetrics were analyzed.

**Results:**

The SAA− and matched SAA+ groups had similar motor performance on the Movement Disorder Society Unified Parkinson's Disease Rating Scale‐Part III (MDS‐UPDRS‐III) and similar cognitive performance on the Montreal Cognitive Assessment (MoCA) at baseline. The proportion with severe hyposmia was 12% for SAA− versus 73% for SAA+ (*P* < 0.001). Per PPMI enrollment criteria all participants were classified as having an abnormal DAT‐SPECT. There were no significant differences in median quantitative DAT‐SPECT measures between groups. The SAA− group showed a higher degree of atrophy in subcortical brain regions including substantia nigra. Longitudinally, 14.3% of SAA− participants had a change in diagnosis versus 0.9% of SAA+ participants.

**Conclusions:**

At baseline, SAA− sPD PPMI participants have a substantially lower rate of hyposmia, but otherwise cannot be readily distinguished from SAA+ participants based on clinical characteristics. However, SAA− participants have a greater degree of subcortical brain atrophy, and approximately one out of seven SAA− participants received a change in diagnosis. © 2026 The Author(s). *Movement Disorders* published by Wiley Periodicals LLC on behalf of International Parkinson and Movement Disorder Society.

Pathologically, Parkinson's disease (PD) is characterized by intra‐neuronal accumulation of alpha‐synuclein (α‐syn) within Lewy bodies and Lewy neurites. Until recently, confirmation of α‐syn aggregation was only feasible via pathologic assessment of post‐mortem brain tissue. As such, clinical trials use established clinical diagnostic criteria,^1^ and when enrolling newly diagnosed PD may supplement with a biomarker of presynaptic dopaminergic neuronal dysfunction such as dopamine transporter‐single photon emission computed tomography (DAT‐SPECT).^2^ While DAT‐SPECT increases diagnostic accuracy, DAT‐SPECT abnormalities are non‐specific for PD,^3^ and clinical trials using these criteria still may enroll a subset of individuals without Lewy‐type pathology.

We have recently introduced a research framework for a biological definition of neuronal synuclein disease (NSD) that includes biomarkers specific for identifying people with Lewy‐type pathology.^4^ One use of this framework could be to increase the positive predictive value of the clinical diagnosis for presence of Lewy‐type pathology early in the disease course when the clinical diagnosis may be less accurate. This framework leverages advances in α‐syn biomarker development, specifically the α‐syn seed amplification assay from cerebrospinal fluid (CSFasynSAA).^5^ CSFasynSAA has emerged as a reliable biomarker for detection of aggregated α‐syn, and CSFasynSAA has been validated as a highly sensitive and specific technique for differentiating those with a clinical diagnosis of PD from healthy control individuals.^6^, ^7^, ^8^

Across multiple cohorts, including the Parkinson's Progression Markers Initiative (PPMI), over 90% of people with sporadic PD (sPD) have positive CSFasynSAA.^9^, ^10^ Notably, amongst people with PD and pathogenic *LRRK2* variants, approximately one‐third have negative CSFasynSAA and an absence of Lewy‐type pathology on post‐mortem autopsy.^9^, ^11^, ^12^ Thus, genetic factors likely play a key role in regulating disease pathogenesis and biomarker results must thus be interpreted in the context of genetic results when available. However, for those individuals without *LRRK2* variants or other identified genetic etiologies for PD it remains unknown how those with clinical sPD with a negative CSFasynSAA (SAA−) differ from those with positive CSFasynSAA (SAA+). Answering this question could clarify the potential utility of the CSFasynSAA assay, and the application of the NSD framework, to clinical trial design. Specifically, if the progression of SAA− individuals differs from those who are SAA+, inclusion of SAA− individuals into clinical trials could introduce heterogeneity that is unrelated to Lewy‐type pathology.

To address this knowledge gap, we aimed to determine whether sPD participants in the PPMI cohort who are SAA− at baseline have distinct clinical and neuroimaging characteristics compared with SAA+ participants. We also sought to evaluate longitudinal disease progression amongst SAA− sPD participants and determine whether the diagnosis of PD in these individuals was revised over time.

## Patients and Methods

### Study Population

Data were from PPMI, a multicenter prospective cohort study. The PPMI study protocol, including biofluid collection techniques and storage processes, have been published elsewhere in detail.^13^ Participants included in the study were those enrolled in the sPD PPMI cohort prior to March 10, 2025, who had CSFasynSAA performed at baseline enrollment. The inclusion criteria for enrollment in the sPD PPMI cohort were: (1) clinical diagnosis of PD, (2) time since clinical diagnosis of 2 years or less, and (3) abnormal DAT‐SPECT based on study criteria which included visual review by at least two nuclear medicine physicians with experience in reading DAT‐SPECT imaging. The PPMI exclusion criteria for enrollment in the sPD PPMI cohort are detailed in full at ppmi-info.org. Of note, treatment with dopaminergic medication within 60 days of enrollment is one of the exclusion criteria. Participants were additionally excluded from this analysis if they had: (1) inconclusive or type II CSFasynSAA result at baseline analysis (see below for details) or (2) were PPMI participants with a pathogenic variant in PD‐associated genes (see below for details).

### CSFasynSAA

CSF analysis was performed at the baseline visit for all participants. Assessment of aggregated α‐syn was performed via a CSFasynSAA completed by Amprion (Amprion Inc., San Diego, CA, USA) as previously described.^9^, ^14^ There are two protocols that have been used in PPMI, namely the Amprion 150 hr assay and the newer Amprion 24 hr assay. The following assay parameters were used to define CSFasynSAA results: Fmax (highest fluorescence intensity), T50 (time to 50% of Fmax), and TTT (time to reach a target relative fluorescence unit threshold). Based on interpretation of these parameters, the following possible results were obtained: SAA−, SAA+, and inconclusive. The positive 24 hr CSFasynSAA shows a distinct type I pattern in PD subjects and type II pattern in individuals with a pathologically confirmed diagnosis of multiple system atrophy (MSA).^14^ Therefore, for participants for whom both the 150 hr and 24 hr CSFasynSAA were performed, results of the 24 hr CSFasynSAA were used to determine CSFasynSAA status. The number of SAA− participants who had the 150 and 24 hr CSFasynSAA protocol and the concordance between assay results is detailed in Table S1. Regarding blinding, an elective return of research information (RORI) PPMI initiative was initiated in December 2023 so some participants and investigators may have been informed of CSFasynSAA results. Future work is needed to determine whether RORI has impacted investigator diagnosis classifications in PPMI.

### Clinical Assessments

Demographic data were collected per the PPMI protocol as previously reported, including age at enrollment, sex, race, Hispanic or Latino ethnicity, and education.^13^ Time since diagnosis at date of enrollment and family history of PD were recorded. The PPMI study includes clinical measures assessing motor performance, non‐motor symptoms, and cognitive function conducted annually. This study included data on Hoehn & Yahr Stage, Movement Disorder Society‐Unified Parkinson's Disease Rating Scale (MDS‐UPDRS)‐Parts I, II, III, and IV scores, percentile score on the University of Pennsylvania Smell Identification Test (UPSIT), Modified Schwab & England Activities of Daily Living score, REM Sleep Behavior Disorder (RBD) screen questionnaire score (RBDSQ), Geriatric Depression Scale (GDS), and Scales for Outcomes in Parkinson's disease‐Autonomic (SCOPA‐AUT), including total SCOPA‐AUT score and subscores in each autonomic domain. For cognitive function, the Montreal Cognitive Assessment (MoCA) score was used as well as investigator diagnosis of cognitive status (normal, mild cognitive impairment, or dementia). Per PPMI protocol, at enrollment sPD participants are not allowed to be on dopaminergic medications. Once participants initiate dopaminergic therapy MDS‐UPDRS‐III is assessed in medication OFF and ON states. The percentage of participants who initiated dopaminergic medications was assessed at the 1‐year follow‐up. For those participants on dopaminergic medications at 1 year, the difference between MDS‐UPDRS‐III scores ON medication and OFF medication was calculated. To assess motor asymmetry, a motor symptom asymmetry index was calculated as follows: lateralized items on MDS‐UPDRS‐III were assessed (items 3.3, 3.4, 3.5, 3.6, 3.7, 3.8, 3.15, 3.16, and 3.17) and the following calculation was made: absolute value of (right side − left side)/(right side + left side). At annual visits the PPMI site investigator makes a clinical determination of the most likely diagnosis. For this study dementia with Lewy bodies was not considered a change in diagnosis as it is a neuronal synuclein disease.

### Genetic Testing

Genotyping methods for the PPMI study are described at ppmi-info.org. Participants with known pathogenic variants identified in the *LRRK2*, *GBA1*, *PRKN*, or *SNCA* genes were excluded from analysis. The following *LRRK2* pathogenic variants were tested for: G2019S, R1441C, R1441G, R1441H, I2020T, and N1437H. Genetic testing for the *LRRK2* G2019S variant was available for 76/80 (95%) of SAA− sPD participants and was negative in all SAA− sPD cases tested. Assessment for the complete set of pathogenic *LRRK2* variants was available for 58/80 (73%) of SAA− participants, and one individual in the SAA− sPD group was found to have a *LRRK2* R1441G variant. The positive *LRRK2* genetic result in that SAA− participant was received after primary analysis was completed and that participant was thus not excluded from the cohort.

### Dopamine Transporter Imaging Analysis

DAT‐SPECT at baseline visit was used to evaluate dopamine dysfunction in SAA− and SAA+ groups. Dopamine dysfunction was assessed quantitatively by the following parameters: lowest putamen‐specific binding ratio (SBR) percent expected for age and sex, mean striatum binding, mean caudate binding, mean putamen binding, and the DAT binding asymmetry index, calculated as previously described.^15^

### Magnetic Resonance Imaging Analysis

Magnetic resonance imaging (MRI) sequences for PPMI are described at ppmi-info.org (https://www.ppmi‐info.org/sites/default/files/docs/PPMI2.0_002_MRI_TOM_Final_v4.0_20221118_FE.pdf). T1‐weighted MRI at baseline visit was used to evaluate atrophy patterns between SAA− and SAA+ sPD groups. Similar to our previous work, deformation‐based morphometry (DBM) was used as the main measure of regional brain atrophy.^16^, ^17^, ^18^ Briefly, all T1‐weighted images were preprocessed including denoising,^19^ non‐uniformity correction,^20^ and intensity normalization. All images were then registered linearly and nonlinearly to MNI‐ICBM‐2009c template. All steps underwent visual quality control and participant scans that did not pass the quality control in any step were excluded from analysis (67/516 scans, ~12.98%), consistent with the PPMI dataset failure rate at 13.10%. Voxel‐wise DBM maps were generated by calculating the determinant of the Jacobian based on the deformation field based on the nonlinear transformations from subject T1‐weighted to the template. These voxel‐wise DBM maps were then averaged using an accurate atlas of 22 subcortical brain regions based on integration of BigBrain and MNI‐ICBM‐2009c template to calculate the regional DBM values for each participant scan for the statistical analysis.^21^ MRI analysis was carried out with the matched subset of subjects based on propensity‐score matching as well as the complete unmatched sample. All neuroimaging results are reported as significant after false discovery rate (FDR) correction for multiple comparisons using a threshold of 0.05.

### Longitudinal Outcomes

Disease progression was assessed using time to domain‐based milestones as described.^22^ This methodology assesses 25 disease milestone metrics across six domains: “walking and balance,” “motor complications,” “cognition,” “autonomic dysfunction,” “functional dependence,” and “activities of daily living.” For each domain the participant was designated as having progressed to meet that domain if they had reached any of the previously defined milestone criteria within that domain. For example the “walking and balance” domain milestones are as follows: walking and balance impairment defined as MDS‐UPDRS item 2.12 response ≥3; freezing of gait defined as MDS‐UPDRS item 2.13 response ≥3 or MDS‐UPDRS item 3.11 response = 4 (ON or OFF medications); gait impairment defined as MDS‐UPDRS item 3.10 response ≥3 (ON or OFF medications); postural instability defined as MDS‐UPDRS item 3.12 response ≥3 (ON or OFF medications); or Hoehn & Yahr stage ≥4. Details on milestone criteria for the other domains can be found as previously reported by Brumm et al.^22^ Other outcomes assessed include Hoehn & Yahr stage ≥3, moderate to severe non‐dyskinesia motor complications defined as a score of ≥3 on MDS‐UPDRS items 4.3 or 4.4, and research diagnosis.

### Neuropathologic Assessment

Autopsy evaluation of post‐mortem brain tissue was available for only one SAA− sPD participant. Neuropathologic assessment was performed as described previously.^23^

### Statistical Analysis

Baseline demographic, clinical, and DAT‐SPECT characteristics are summarized for SAA− and SAA+ sPD groups, using median (interquartile range [IQR]) and/or mean (standard deviation [SD]) for continuous measures and frequency (percentage) for categorical measures. Differences in baseline characteristics between groups were assessed using the Wilcoxon rank‐sum test for continuous variables, and chi‐square (or Fisher's exact test when at least one expected cell count is below 5) for categorical variables.

For longitudinal survival analyses, Kaplan–Meier curves were generated for each endpoint as defined by Brumm et al.^22^: any progression milestone, six domain milestones; Hoehn & Yahr stage ≥3; MDS‐UPDRS item 4.3 or 4.4 ≥ 3. These curves are presented by group over a 3‐year period from enrollment, with at‐risk tables and log‐rank *P*‐value. Participants who did not experience an event during the follow‐up period were censored at the time of their last assessment within 3 years. Participants who had already reached any progression milestone or either of the two other survival outcomes at the baseline visit were excluded from analysis.

To account for age differences at enrollment between SAA− and SAA+ sPD groups, a propensity‐score matching approach was employed where SAA+ sPD participants were matched to the SAA− sPD group based on age, sex, and time since diagnosis. The matching process utilized a caliper width of 0.25 times the standard deviation of the logit of the propensity score with an optimal fixed‐ratio matching procedure designed to maximize the number of matched units while minimizing the standardized mean differences. One SAA− participant was excluded from matched analysis due to missing data on time since clinical diagnosis.

Statistical analyses were performed using SAS v9.4 (SAS Institute Inc., Cary, NC, USA; sas.com; RRID:SCR_008567). Sankey diagrams were prepared using the “ggsankey” package in R (version 4.1.1; R Core Team 2024).

## Data Sharing

Data used in preparation of this article were obtained on March 10, 2025 from the PPMI database (www.ppmi‐info.org/access‐data‐specimens/download‐data), RRID:SCR_006431. For up‐to‐date information on PPMI, visit www.ppmi-info.org. This analysis was conducted by the PPMI Statistics Core and used actual dates of activity for participants, a restricted data element not available to public users of PPMI data. Statistical analysis codes used to perform the analyses in this article are shared on Zenodo (10.5281/zenodo.15238103).

## Results

### Sample Characteristics

Of the total sPD PPMI participants 80 were SAA− compared with 856 who were SAA+ at baseline. Of the 80 SAA− participants, repeat CSFasynSAA data at follow‐up visits was available for 26. Twenty‐two (84.6%) of these SAA− participants remained SAA− on follow‐up while 4 (15.4%) had a type II result on follow‐up. None of the SAA− participants had a positive type I SAA on follow‐up. Of the total 856 SAA+ sPD PPMI participants, repeat CSFasynSAA at a follow‐up visit was available for 309 participants. Of these, 292 (94.5%) of the SAA+ participants remained SAA+ at follow‐up, 8 (2.6%) had a negative CSFasynSAA result, 5 (1.6%) had a type‐II CSFasynSAA result, and 4 (1.3%) had an inconclusive CSFasynSAA.

### Baseline Demographic Characteristics of SAA− sPD Participants

The median age at enrollment for the SAA− group was 66.8 years (IQR 61.7–73.1) compared with 63.9 years (IQR 57.2–69.9) in the SAA+ group (*P* = 0.001) (Table S2). The median time since diagnosis and distribution of sex was comparable between groups (Table S2).

Due to the statistically significant between‐group age difference, subsequent analyses used a reduced dataset matched for age, sex, and time since diagnosis, as described in the Methods section. The number of participants included in the matched dataset was n = 79 SAA− participants and n = 237 SAA+ participants. Of note, one SAA− participant was excluded from matched analysis due to missing data on time since clinical diagnosis.

### Baseline Clinical Characteristics

**FIG. 1:**
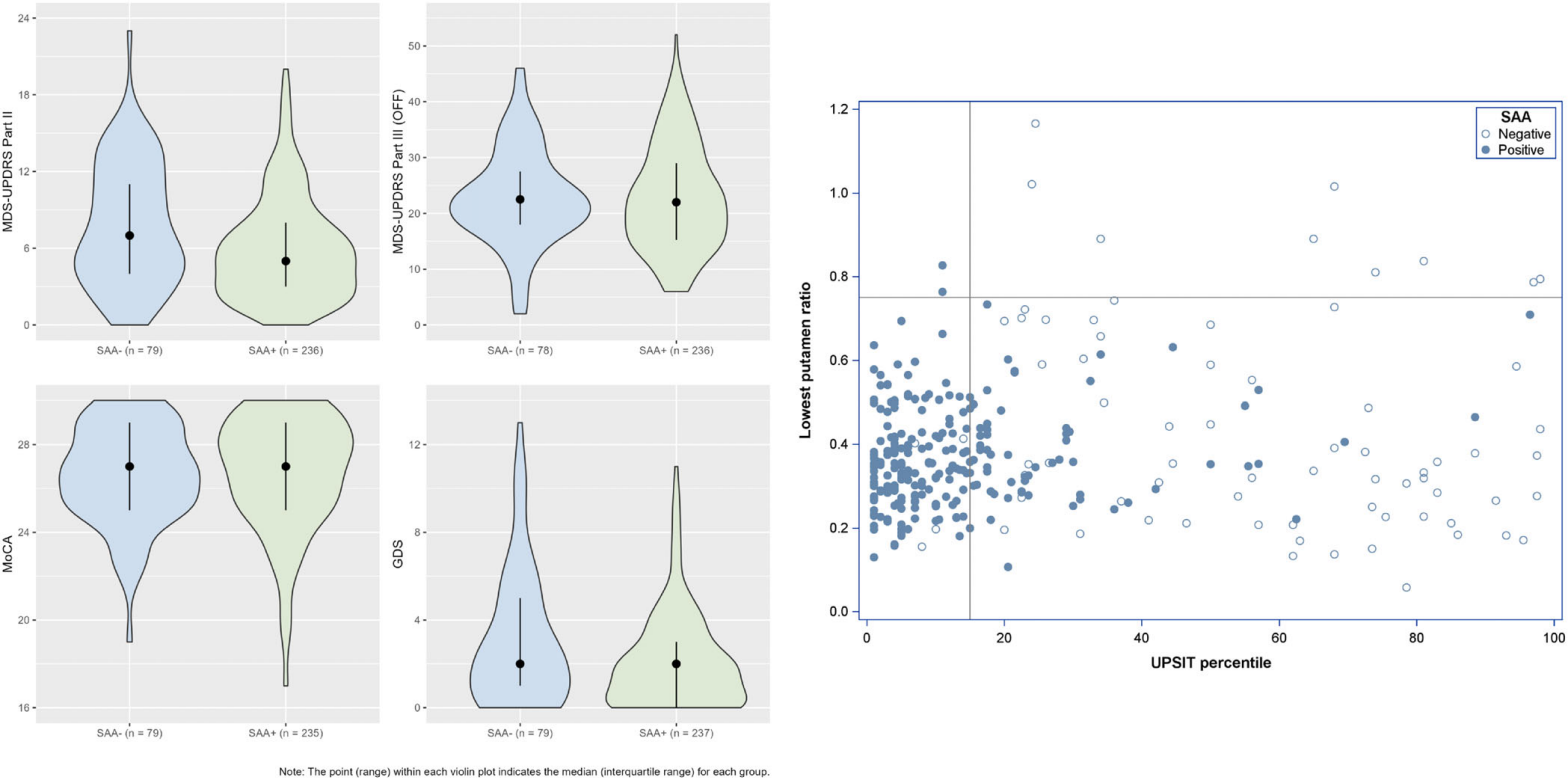
Baseline characteristics of seed amplification assay negative (SAA−) and positive (SAA+) sporadic Parkinson's disease (sPD) participants. (A) Violin plots of selected clinical characteristics measured at the baseline assessment are presented for SAA− and SAA+ participants. The number of SAA− and SAA+ participants for whom data is available is indicated on the *x*‐axis. For each plot the point indicates the median and the range indicates the interquartile range. (A) Median score on the Movement Disorder Society‐Unified Parkinson's Disease Rating Scale‐Part II (MDS‐UPDRS‐II) (*P* = 0.003). (B) Median score on the MDS‐UPDRS‐III in the OFF state (*P* = 0.648). (C) Median score on the Montreal Cognitive Assessment (MoOCA) (*P* = 0.531). (D) Median score on the Geriatric Depression Scale (GDS) (*P* = 0.025). (B) The association between olfaction, dopamine transporter binding, and cerebrospinal fluid alpha‐synuclein seed amplification assay (CSFasynSAA) results is shown. Olfaction as indicated by percentile score on the University of Pennsylvania Smell Identification Test (UPSIT) is shown on the *x*‐axis and dopamine transporter (DAT) scan lowest putamen‐specific binding ratio is shown on the *y*‐axis. SAA− participants are represented with an open circle and SAA+ participants are represented with a closed circle. [Color figure can be viewed at wileyonlinelibrary.com]

Baseline demographic and clinical characteristics of the matched SAA− (n = 79) and SAA+ (n = 237) sPD groups are summarized in Table 1 and Table S3. Notably, the median percentile score on the UPSIT was 55.0 (IQR 26.0–78.5) for the SAA− sPD group compared with 8.0 (IQR 4.0–16.0) for SAA+. The percentage of participants with an UPSIT score less than or equal to the 15th percentile was 12% for SAA− versus 73% for SAA+. The SAA− group had a higher median score on the MDS‐UPDRS‐II (*P* = 0.003), but there was no significant difference in MDS‐UPDRS‐III scores in the OFF state (*P* = 0.648) (Fig. 1A). Analysis of tremor subscores demonstrated a lower rest tremor subscore in the SAA− group compared with SAA+ (*P* = 0.002). Regarding non‐motor features, there was no significant difference between groups in cognitive performance on the MoCA (*P* = 0.531) (Fig. 1A) or autonomic features as assessed by median SCOPA‐AUT score (*P* = 0.151).

**TABLE 1:** Demographic and baseline characteristics of matched seed amplification assay negative (SAA−) and positive (SAA+) sporadic Parkinson's disease participants.

Variable	SAA− sPD (N = 79^a^)	SAA+ sPD (N = 237)	*P*‐value^b^
Age at enrollment, years, median (IQR)	66.7 (61.3–73.3)	67.4 (62.1–72.5)	0.959
Male sex, n (%)	50 (63%)	150 (63%)	1.000
Family history of PD, n (%)			0.752
First‐degree family with PD	10 (13%)	37 (16%)	
Non‐first‐degree family with PD	9 (11%)	30 (13%)	
No family with PD	60 (76%)	170 (72%)	
Time since diagnosis at enrollment, median (IQR)	0.5 (0.3–0.8)	0.5 (0.3–0.8)	0.369
Duration of follow‐up from BL, years, median (IQR)	1.9 (1.0–3.0)	2.1 (1.0–6.0)	0.060
Hoehn & Yahr stage, n (%)^c^			0.201^c^
1	19 (24%)	75 (32%)	
2	59 (75%)	161 (68%)	
3	1 (1%)	1 (<1%)	
UPSIT percentile, median (IQR)	55.0 (26.0–78.5)	8.0 (4.0–16.0)	<0.001
UPSIT ≤ 15th percentile, n (%)	9 (12%)	171 (73%)	<0.001
MDS‐UPDRS‐I, median (IQR)	6.0 (4.0–11.0)	5.0 (3.0–8.0)	0.069
MDS‐UPDRS‐II, median (IQR)	7.0 (4.0–11.0)	5.0 (3.0–8.0)	0.003
MDS‐UPDRS‐III (OFF), median (IQR)	22.5 (18.0–27.0)	22.0 (15.5–29.0)	0.648
Motor symptom asymmetry index (OFF), median (IQR)	0.40 (0.20–0.60)	0.52 (0.32–0.81)	0.001
Tremor score (OFF), median (IQR)	4.0 (2.0–7.0)	5.0 (3.0–7.0)	0.075
Rest tremor subscore (OFF), median (IQR)	2.0 (0.0–4.0)	4.0 (2.0–5.0)	0.002
MDS‐UPDRS Total Score (OFF), median (IQR)	39.0 (28.0–48.0)	32.0 (25.0–43.0)	0.020
MoCA, median (IQR)	27.0 (25.0–29.0)	27.0 (25.0–29.0)	0.531
Cognitive categorization, n (%)			0.244
Normal	54 (83%)	150 (89%)	
Mild cognitive impairment	11 (17%)	19 (11%)	
GDS, median (IQR)	2.0 (1.0–5.0)	2.0 (0.0–3.0)	0.025
SCOPA‐AUT, median (IQR)	11.0 (7.0–14.0)	9.0 (6.0–13.0)	0.151
Lowest putamen ratio, median (IQR)	0.35 (0.23–0.60)	0.35 (0.29–0.43)	0.736
Mean striatum binding, median (IQR)	1.29 (1.03–1.83)	1.40 (1.18–1.67)	0.557
Mean caudate binding, median (IQR)	1.87 (1.45–2.33)	1.93 (1.66–2.35)	0.188
Mean putamen binding, median (IQR)	0.84 (0.56–1.39)	0.84 (0.68–1.05)	0.704
DAT binding asymmetry index, median (IQR)	0.12 (0.07–0.20)	0.15 (0.08–0.25)	0.169
Lowest putamen ratio ≥ 0.75, n (%)	9 (12%)	2 (0.9%)	<0.001

*Note*: Missing data (if more than 5): Cognitive categorization n = 82 (consistent data collection on this variable began after 2020); DAT scan measures (lowest putamen ratio, mean binding, asymmetry index) n = 6.

Abbreviations: SAA, seed amplification assay; sPD, sporadic Parkinson's disease; IQR, interquartile range; BL, baseline; UPSIT, University of Pennsylvania Smell Identification Test; MDS‐UPDRS, Movement Disorder Society‐Unified Parkinson's Disease Rating Scale; OFF, off medication; MoCA, Montreal Cognitive Assessment; SCOPA‐AUT, Scales for Outcomes in Parkinson's Disease‐Autonomic; GDS, Geriatric Depression Scale; DAT dopamine transporter.

^a^ One SAA− sPD participant excluded from matched analysis due to missing disease duration.

^b^ Comparisons by SAA status used chi‐square or Fisher's exact tests for categorical variables and Wilcoxon rank‐sum tests for continuous variables.

^c^ For the purposes of comparisons, Hoehn & Yahr stage was dichotomized as stage 1 versus ≥2.

### Baseline DAT‐SPECT Metrics

All of the participants were classified as having an abnormal DAT‐SPECT at baseline as per PPMI enrollment criteria, which included visual inspection with or without quantitative analysis. There were no significant differences between matched SAA− and SAA+ sPD groups in the following quantitative DAT‐SPECT imaging metrics at baseline assessment: median lowest putamen SBR, mean striatum binding, mean caudate binding, mean putamen binding, and the DAT binding asymmetry index (Table 1). However, while there was no difference between median lowest putamen ratio between groups, we found that 9/75 (12.0%) of the matched SAA− participants had a lowest putamen ratio ≥ 0.75 compared with 2/235 (0.9%) of the matched SAA+ participants (*P* < 0.001). Four participants in the matched SAA− group and two participants in the matched SAA+ group had missing lowest putamen ratio at baseline. The association between dopamine transporter binding, olfaction, and CSFasynSAA results is shown in Figure 1B.

### Baseline Structural MRI Analysis

**FIG. 2:**
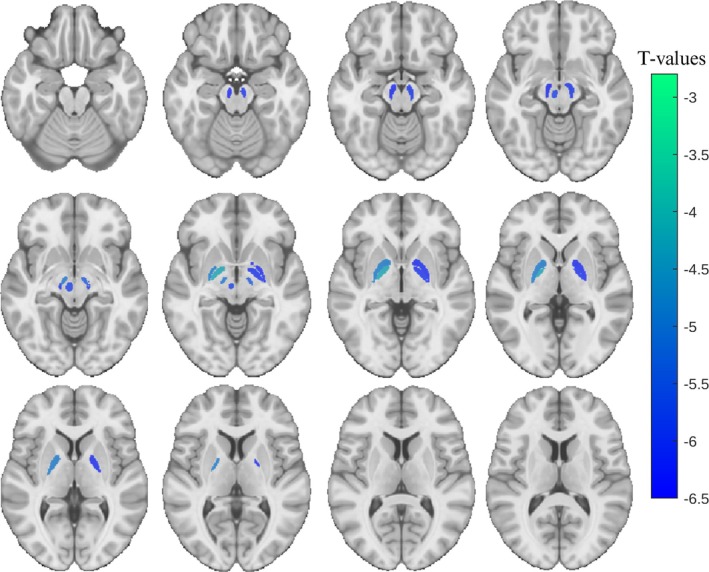
Cross‐sectional brain atrophy map of the seed amplification assay negative (SAA−) sporadic Parkinson's disease (sPD) group. T‐statistic maps (false discovery rate [FDR]‐corrected *P* < 0.05) show regions where the SAA− group exhibited significantly greater atrophy compared with the matched seed amplification assay positive (SAA+) group. Results are overlaid on axial slices of the MNI template, ranging from *z* = 58 (top left) to *z* = 91 (bottom right) in 3 mm increments. DBM, deformation based morphometry. The colormap indicates the T‐values from the SAA− group contrasted between SAA− and SAA+ participants from the linear model controlling for age and sex. [Color figure can be viewed at wileyonlinelibrary.com]

Compared with the propensity‐score matched SAA+ group, SAA− sPD participants had a significantly higher degree of atrophy in 9 of 22 subcortical regions including bilateral substantia nigra, subthalamic nucleus, globus pallidus interna and externa, and left red nucleus (FDR corrected *P* < 0.05) (Fig. 2, Table S4). While not significant, the remaining 13 regions except for the right caudate showed a trend towards higher atrophy. To ensure the robustness of our findings, we repeated the analysis on the full unmatched sample, including age and sex as covariates. The results were consistent, except for the left putamen, which reached statistical significance in the full‐sample analysis (Table S5).

### Longitudinal Analysis

Median follow‐up from baseline was 1.9 years for the SAA− group. At 2 years there were data available for 53% of SAA− sPD participants; 39% of SAA− participants had not yet reached the 2‐year enrollment timepoint, 4% were lost to follow‐up or were overdue for their 2‐year visit, and 5% withdrew from the study (Table S6).

**FIG. 3:**
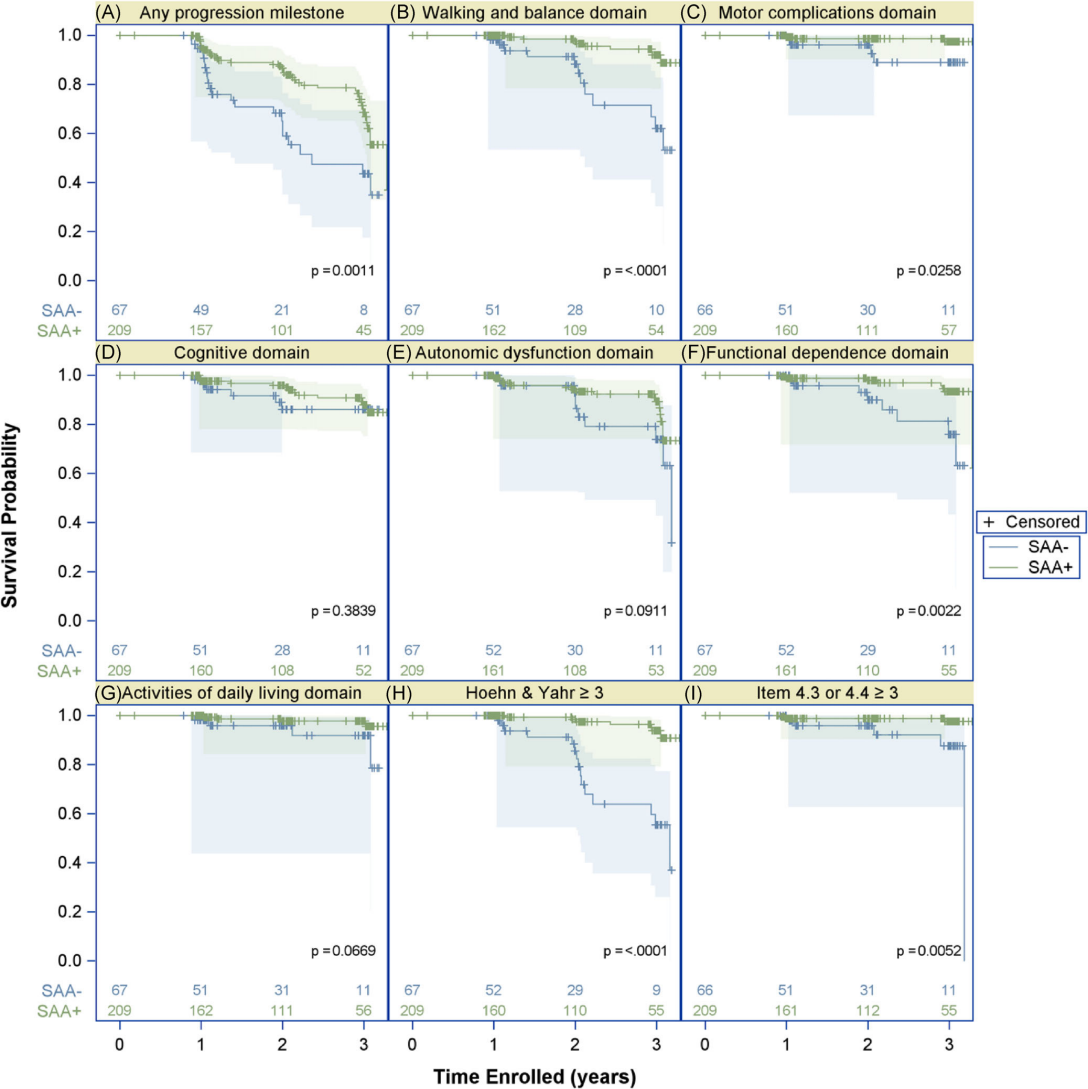
Time to reach disease progression milestones in matched seed amplification assay negative (SAA−) and positive (SAA+) sporadic Parkinson's disease (sPD). Kaplan–Meier curves are presented for time to reach disease progression milestones in the SAA− and SAA+ matched groups over a 3‐year time period. The number of SAA− and SAA+ participants for whom data is available at each annual time point is indicated. The endpoints analyzed include: (A) time to reach any of the six domain progression milestones defined by Brumm et al.,^22^ (B) walking and balance domain milestones, (C) motor complications domain milestones, (D) cognitive domain milestones, (E) autonomic dysfunction domain milestones, (F) functional independence domain milestones, (G) activities of daily living domain milestones, (H) Hoehn & Yahr stage ≥3, and (I) moderate to severe non‐dyskinesia motor complications defined as a score of ≥3 on the Movement Disorder Society‐Unified Parkinson's Disease Rating Scale (MDS‐UPDRS) item 4.3 or 4.4 which measure time in the OFF state and functional impact of motor fluctuations, respectively. [Color figure can be viewed at wileyonlinelibrary.com]

Compared with the propensity‐score matched SAA+ sPD group, the SAA− group had a faster time to reach any domain‐based disease milestones (Fig. 3). The most notable difference in attainment of milestones was in the proportion of participants who met the walking and balance milestones, which includes complications such as freezing of gait and postural instability. The walking and balance milestones were met by 13% of SAA− participants within 2 years compared with 3% of SAA+. For those SAA− participants who reached walking and balance milestones the most common milestone met was postural instability (Table S7).

### Dopaminergic Treatment

At 1‐year follow‐up 33/66 (50%) of SAA− participants and 126/202 (62.4%) of matched SAA+ participants had initiated dopaminergic treatment. Among the subset of matched participants who were on dopaminergic treatment, the median difference between MDS‐UPDRS‐III (ON) and MDS‐UPDRS‐III (OFF) at year 1 was not significant (−7.5 [−11, 0; n = 22] in the SAA− group and − 6.0 (−11, 0; n = 81) in the SAA+ group (*P* = 0.71)].

### Diagnostic Change over Time

**FIG. 4:**
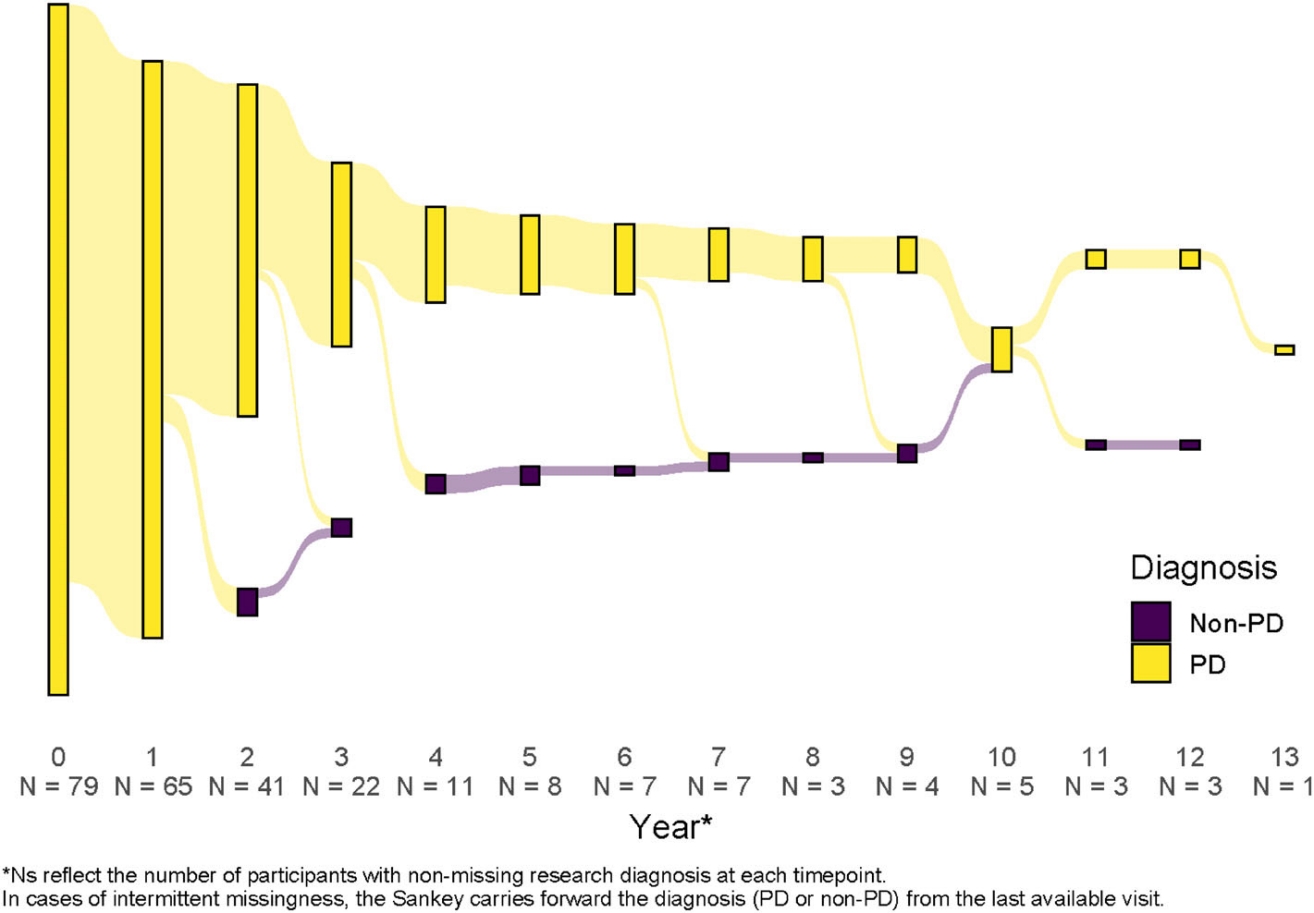
Sankey diagram of primary research diagnosis in seed amplification assay negative (SAA−) sporadic Parkinson's disease (sPD) participants at annual visits. A Sankey diagram is presented indicating the primary research diagnosis recorded at each annual follow‐up visit for the SAA− sPD group. Yellow indicates a diagnosis of PD and purple indicates any other diagnosis that is not PD. The height of the bar at each year is proportional to the number of participants in each diagnosis category. For each annual timepoint the number of SAA− participants for whom research diagnosis information was available is indicated on the *x*‐axis. [Color figure can be viewed at wileyonlinelibrary.com]

Amongst the 80 SAA− sPD participants, 77 had at least one follow‐up visit and of these participants 11 (14.3%) had a change in research diagnosis at a follow‐up visit (Fig. 4). By contrast, of the 237 matched SAA+ sPD group there were 231 who had at least one follow‐up visit, and only 2/231 (0.9%) had a change in diagnosis to MSA and “other primary parkinsonism,” respectively.

In the SAA− group, six participants (7.8%) had their research diagnosis changed to MSA. The other changed diagnoses were progressive supranuclear palsy (PSP), corticobasal syndrome (CBS), primary progressive freezing of gait, and prodromal synucleinopathy, each in one individual participant. In addition, one participant was deemed not to have PD or another neurologic disorder. Amongst the six participants for whom the diagnosis was changed to MSA, repeat CSFasynSAA data were available for two participants, one of whom remained SAA− and the other had a type II MSA‐like result at 1‐year follow‐up. To assess whether elective RORI may have impacted investigator‐determined diagnostic classifications, we evaluated the dates in which SAA− participants accessed their CSFasynSAA results relative to the date of the change in diagnosis. We found that of the SAA− participants who had a change in research diagnosis, none of these participants had accessed the CSFasynSAA results prior to the date of the change in diagnosis. To determine whether participants with a change in diagnosis impacted analysis of longitudinal disease progression we performed assessment of time to reach domain‐based milestones with exclusion of the 11 SAA− participants who had a change in research diagnosis. With these participants excluded, the SAA− group still had a faster time to reach any domain‐based disease milestone (*P* = 0.0023), and a faster time to walking and balance milestones (*P* = 0.0007) (Fig. S1).

### Pathologic Assessment

Post‐mortem pathologic assessment of brain tissue was available for one of the SAA− sPD participants. The research diagnosis for this individual was changed from PD to MSA at year 4 of follow‐up. The participant died 7 years following enrollment in the study. Post‐mortem brain tissue examination demonstrated glial cytoplasmic inclusions of α‐syn consistent with a diagnosis of MSA‐P.

## Discussion

In this study, we assessed clinical and neuroimaging characteristics of sPD participants in the PPMI cohort who lacked evidence of α‐syn aggregation in the CSF and compared these participants with SAA+ sPD participants matched by key demographic characteristics. We found that there were several clinical features that differed between the two groups. Foremost amongst these was hyposmia, with 73% of SAA+ participants performing at or less than the 15th percentile on the UPSIT scale while only 12% of SAA− sPD participants were hyposmic by this parameter. This finding is consistent with prior reports of high correlation between hyposmia and α‐syn pathology.^9^, ^24^ However, the SAA− and SAA+ sPD groups had similar baseline motor features and cognitive assessments and thus the data suggest that aside from hyposmia, standard clinical assessments alone at the time of clinical diagnosis are inadequate to identify individuals who may lack α‐syn pathology. The strong concordance between hyposmia and CSFasynSAA positivity both in our analysis and in previous reports from the literature suggests that testing for hyposmia could be considered as a potential clinical marker for assessing the likelihood of α‐syn pathology. Notably, although RBD is another common prodromal feature of synucleinopathies, we did not see a difference in median score on the RBD screening questionnaire between the SAA− and SAA+ groups.

Using MRI deformation‐based morphometry, the SAA− participants showed significantly greater atrophy at a group level in multiple subcortical regions, including the substantia nigra, subthalamic nucleus, globus pallidus, putamen, and red nucleus. These differences were significant after matching for age, suggesting that the observed atrophy is not solely age‐related. The underlying cause of the increased atrophy of subcortical brain regions in the SAA− sPD group is unknown, but one hypothesis is that a subset of these individuals may in fact have atypical parkinsonian syndromes despite having a clinical diagnosis of sPD.

In our study, all individuals were classified as having a positive DAT‐SPECT scan at baseline as this is part of the enrollment criteria into the sPD PPMI cohort. When evaluating the SAA− and SAA+ sPD groups as a whole we found no differences between the SAA− and SAA+ sPD groups in median quantitative DAT‐SPECT metrics. Therefore SAA− and SAA+ sPD individuals cannot be readily distinguished based on DAT‐SPECT results alone. However, notably a subset of individuals had a lowest putamen ratio ≥ 0.75, which is used as a quantitative threshold for normal, despite having been classified as having a positive DAT scan by visual interpretation and this proportion was higher in the SAA− sPD group. This suggests that there can be discrepancies between visual interpretation of DAT‐SPECT and quantitative metrics, and that in clinical trial design detailed quantitative DAT‐SPECT assessment in conjunction with fluid biomarker data could allow for more precise biological characterization of individuals with parkinsonism.

On longitudinal assessment, progression to clinically meaningful milestones appears to be faster in SAA− individuals, but confirmation of the rate of disease progression in SAA− sPD participants will require future longer longitudinal follow‐up given the limitations of our small number of SAA− participants with long‐term follow‐up data. The SAA− individuals had a notably higher rate of diagnostic change compared with SAA+ sPD individuals. This suggests that CSFasynSAA status could be important to consider in clinical trial design, particularly for α‐syn targeting therapies. Notably, prior studies of the stability of the diagnosis of PD have shown high rates of change in diagnosis.^25^, ^26^, ^27^ But our finding that 14.3% of SAA− sPD participants had a change in research diagnosis compared with only 0.9% of SAA+ sPD participants suggests that at least within the PPMI cohort CSFasynSAA status significantly impacts the stability of the PD diagnosis. This finding was not impacted by knowledge of CSFasynSAA status since we confirmed that those SAA− individuals with a change in diagnosis had not accessed CSFasynSAA results prior to the research diagnosis change. It is important to stress that the results of our study should not be used to draw conclusions on the utility of CSFasynSAA in a clinical context as regards making an initial diagnosis for individuals with parkinsonism, since by design our study was limited to individuals classified as sPD in the PPMI research study, all of whom had DAT‐SPECT imaging and met criteria for an sPD diagnosis. Thus, our cohort is not representative of the breadth of parkinsonian patients a clinician would encounter in the clinic.

When interpreting the study results, we consider potential causes of negative CSFasynSAA, including: (1) a true‐negative in an individual with a clinical syndrome indistinguishable from sPD, (2) a false‐negative in an individual with sPD and Lewy body pathology, and (3) a true‐negative in an individual who was erroneously diagnosed with sPD but actually has an alternative pathology, such as a 4‐repeat (4R) tauopathy or glial cytoplasmic inclusions. To this last consideration, 14.3% of SAA− sPD participants had a change in research diagnosis on follow‐up. However, a significant proportion of the SAA− sPD participants have not yet had extended longitudinal assessment and so longer follow‐up will be required to determine if additional SAA− participants receive a change in diagnosis over time. Given the possibility that some of the SAA− sPD cohort may in fact have atypical parkinsonisms, comprehensive assessment of fluid biomarkers in the SAA− sPD group is an important area for future studies. In particular, emerging evidence indicates that assessment of neurofilament light chain in serum and CSF and evaluation of tau biomarkers could both play a role in differentiating between PD and atypical parkinsonisms.^28^, ^29^, ^30^, ^31^ Ultimately, post‐mortem pathologic assessment will be required to determine the proportion of SAA− participants who were misdiagnosed as sPD and indeed have alternative neuropathologic diagnoses such as 4R‐tauopathies, glial cytoplasmic inclusions, or vascular parkinsonism.

Regarding the possibility of false‐negative CSFasynSAA results, for the subset of SAA− sPD participants for whom repeat CSF analysis was available on follow‐up 85% remained SAA−, 15% had a change to the type II MSA‐like CSFasynSAA result, and no participants had a change to type I SAA+. Therefore, the fairly high consistency of the results is evidence in support of a true‐negative result. The SAA+ sPD group also had a high rate of consistency in CSFasynSAA with 94.5% remaining SAA+ on follow‐up. The one SAA− participant with autopsy confirmation of glial cytoplasmic inclusions, and no Lewy body pathology, also supports this conclusion. However, additional neuropathologic concordance data are necessary to interpret a SAA− result in sPD as our data do not confirm that SAA− status in parkinsonian individuals negates the possibility that they have Lewy‐type pathology at autopsy. Therefore, we do not purport that this study should be extrapolated to the clinical setting and do not suggest that SAA− parkinsonian patients be told they do not have a Lewy body disease based on a biomarker alone. Rather, clinicians should continue to use all of the clinical and biological data, including biomarkers when available, to make the best diagnosis.

The median age at disease onset for the SAA− sPD group was significantly higher than the SAA+ group and we performed age‐matching to account for the potentially confounding effects of age. However, the older age in the SAA− group may in itself be biologically relevant as older individuals may be more likely to have non‐Lewy body pathologies including 4R‐tauopathies such as PSP or corticobasal degeneration.^32^, ^33^ In addition to identifying individuals with pathologic α‐syn, there is an urgent need for development of biomarkers for other pathologies to improve diagnostic accuracy, assess copathology, and improve clinical trial design.

In this study, individuals with pathogenic variants in *LRRK2*, *GBA1*, *PRKN*, and *SNCA* were excluded as they were not classified as sporadic. Notably, amongst people with LRRK2‐PD approximately one‐third lack α‐syn‐containing Lewy body pathology in post‐mortem brain tissue,^11^, ^12^ and 32.5% have negative CSFasynSAA results.^9^ Similarly, PD patients with pathogenic *PRKN* variants often lack neuronal α‐syn on brain autopsy.^34^, ^35^ Since some genetic forms of PD lack pathologic α‐syn, it is possible that a subset of the SAA− participants in this study may have an as yet unrecognized genetic cause of PD resulting in a clinically indistinguishable, yet biologically distinct disease process.

### Limitations and Future Studies

A major limitation of this study is the limited long‐term follow‐up of SAA− sPD participants. The PPMI study will continue longitudinal follow‐up to characterize symptom progression and diagnostic change in the SAA− sPD group. Another factor impacting interpretation of this study is the relatively small number of SAA− sPD participants, which is reflective of the high rate of CSFasynSAA detection in sPD. An additional limitation is that while 76/80 (95%) of SAA− participants were confirmed to be negative for the *LRRK2* G2019S variant, complete genetic testing for the full list of *LRRK2* pathogenic variants was only available for 73% of SAA− participants. One *LRRK2‐*positive SAA− case was included in the cohort since the genetic result was received after primary analysis was already complete. But given that it was only a singular participant, the inclusion of this individual in our dataset does not affect our overall results or conclusions. The rate of *LRRK2* positivity amongst individuals with PD is approximately 2.4%,^36^ and thus amongst the 22 SAA− participants without complete genetic testing it is unlikely that any individuals have a pathogenic *LRRK2* variant. Even for those individuals for whom *LRRK2* testing was complete, it is possible that some individuals could have a *LRRK2* variant of undetermined significance that has not been classified as pathogenic. We anticipate that at least a subset of SAA− sPD cases may have genetic modifiers that could explain the lack of α‐syn aggregation in CSF and thus future work is needed to perform detailed genetic analysis of the SAA− sPD group to further investigate the influence of non‐pathogenic genetic modifiers.

One additional factor impacting study interpretation is that the SAA− sPD group is heterogeneous with a subset of individuals likely falling into distinct disease categories, including those with MSA, PSP, or CBD, who were misdiagnosed as sPD. Even amongst the SAA+ sPD group it is possible that a very small number of individuals could in fact have atypical parkinsonisms, and notably α‐syn copathology can be observed in 4R‐tauopathies.^10^ In this study we did not include biomarker data regarding copathologies such as tau or more general biomarkers of neurodegeneration such as neurofilament light chain because these data are not yet available for the full cohort Thus, future work will involve a comprehensive assessment of additional fluid biomarkers in the SAA− sPD participants.

## Conclusions

SAA− sPD PPMI participants have a substantially lower rate of hyposmia compared with SAA+ participants but otherwise cannot be readily distinguished by baseline clinical characteristics. However, SAA− participants have a higher degree of atrophy in subcortical brain regions and a higher proportion of SAA− participants had a change in diagnosis compared with SAA+ participants. These data suggest that assessment of CSFasynSAA, or alternative validated α‐syn biomarkers, should be considered in sPD clinical trials in which inclusion of individuals unlikely to have Lewy body pathology may confound trial data. Further longitudinal follow‐up of the SAA− sPD PPMI participants will be critical to more fully assess disease progression over time in this group.

## Author Roles

(1) Research Project: A. Conception, B. Organization, C. Execution; (2) Statistical Analysis: A. Design, B. Execution, C. Review and Critique; (3) Manuscript Preparation: A. Writing of the First Draft, B. Review and Critique.

S.M.B.: 1A, 1B, 1C, 2A, 2C, 3A, 3B.

J.P.: 1A, 1B, 1C, 2A, 2C, 3B.

S.H.C.: 1B, 1C, 2A, 2B, 2C, 3B.

D.‐E.L.: 1B, 1C, 2A, 2B, 2C, 3B.

S.‐M.F.: 1A, 1C, 2B, 2C, 3B.

Y.Z.: 1A, 1C, 2B, 2C, 3B.

P.G.: 1C, 2C, 3B.

G.M.R.: 1C, 2C, 3B.

H.A.: 1C, 2C, 3B.

R.M.: 1C, 2C, 3B.

U.J.K.: 1A, 1B, 1C, 2C, 3B.

K.N.H.N.: 1C, 2C, 3B.

A.S.: 1A, 2C, 3B.

C.M.T.: 1A, 2C, 3B.

T.F.T.: 1A, 2C, 3B.

T.F.: 1A, 2C, 3B.

L.M.C.: 1A, 1B 2C, 3B.

B.M.: 1A, 2C, 3B.

K.M.M 1A, 2C, 3B.

D.G.: 1A, 2C, 3B.

C.S.C.: 1A, 2A, 2C, 3B.

R.D.D.: 1A, 2C, 3B.

E.G.B.: 1A, 2C, 3B.

R.N.A.: 1A, 2C, 3B.

D.W.: 1A, 2C, 3B.

K.M.: 1A, 1B, 2C, 3B.

T.S.: 1A, 1B, 2C, 3B.

P.G.L.: 1A, 2C, 3B.

N.P.: 1A, 1B, 2A, 2C, 3B.

K.L.P.: 1A, 1B, 2A, 2C, 3B.

## Financial Disclosures of All Authors (for the Preceding 12 Months)

S.M.B receives funding from the National Institutes of Health/National Institute of Neurological Disorders and Stroke (NIH/NINDS) and The Michael J. Fox Foundation for Parkinson's Research (MJFF). She has received travel expense reimbursement from the Parkinson Study Group and MJFF. J.P. has no disclosures. S.H.C, is funded by grants from MJFF the Michael J Fox Foundation for Parkinson's Research. D.‐E.L. is funded by grants from MJFF. S.‐M.F. has no disclosures. Y.Z. has received research funding from the Fonds de recherche du Québec—Santé Chercheurs boursiers et chercheuses boursières en Intelligence artificielle, Natural Sciences and Engineering Research discovery grant, and Canadian Institutes of Health Research. P.G. has no disclosures. G.M.R. has no disclosures. H.A. has received a PhD scholarship from Fonds de la Recherche du Québec—Santé and Parkinson's Canada. R.M. has no disclosures. U.J.K. is supported by research grants from NIH, Parekh Center for Interdisciplinary Neurology. U.J.K. is on SABA for Amprion, Inc. and NurrON. He is a consultant for UCB, Lundbeck, and HanAll. K.N.H.N. receives grant funding from MJFF, the Alzheimer's Association, the National Collegiate Athletic Association, the U.S. Department of Defense, and the NIH‐National Institute on Aging (NIA). A.S. has been a consultant to the following companies in the past year: Acadia, Eli Lilly and Co, Neurocrine, Theravance, Cerevance, Spark/Roche, Boerhinger‐Ingelheim, Wave Life Sciences, Inhibikase, Prevail, Mitzubishi, and Alertity Therapeutics. He has served on data safety monitoring boards (DSMBs) for the Huntington Study Group and The Healey ALS Consortium (Massachusetts General Hospital). He has received grant funding from MJFF, NIA, and NINDS. C.M.T. declares consultancies for Neurocrine, Praxis (advisory board via the International Parkinson's and Movement Disorder Society), Jazz/Cavion, Roche Genentech. and Bial. C.M.T. also declares grant support to her institution from MJFF, NIH, Gateway LLC, Department of Defense, Roche Genentech, and the Parkinson Foundation. T.F.T. has received research support from NIH, MJFF, the Parkinson Foundation, and Eli Lilly and Company. Dr Tropea serves as a clinical trial advisory board member for Bial, has received travel expense reimbursement from the Parkinson Study Group, The Michael J Fox Foundation and the Parkinson Foundation, and speakers fees from Catalyst Medical Education. T.F. receives funding from NIH and MJFF and serves on several advisory committees for NIH‐funded studies (with <US$1000 each). L.M.C. Financial disclosure: consulting fees and research support from MJFF. COI: none. B.M. has received honoraria for consultancy and/or educational presentations from GE, Bial, Roche, Biogen, AbbVie, Desitin, and Amprion. B.M. is member of the executive steering committee of the Parkinson Progression Marker Initiative of MJFF and has received research funding from Aligning Science Across Parkinson's disease (ASAP, CRN), the German Parkinsonstiftung, and MJFF. COI: none. K.M.M.: Consultant for Axial Therapeutics, Asceneuron, Calico, Hanall, JnJ, MJFF, Nitrase Therapeutics, NuraBio, NRG Therapeutics, Rome Therapeutics, Schrodinger, Ventyx, and private equity companies. Serves on the BoD for Envisagenics, Retromer Tx; serves on the SAB(scientific advisory board) for Axial, Nitrase, NRG, Sinopia, Vanqua; received research funding support from MJFF and honoraria from ASAP. D.G. has received grant support from NIH and MJFF, and is a consultant for Eisai, Lilly, Artery Therapeutics, and Cognition Therapeutics. C.S.C. receives funding from the NIH/NINDS, MJFF, and PCORI. R.D.D. receives research funding from MJFF and the Veterans Affairs Office of Rural Health. E.G.B. has received grant support for research from MJFF, NIH, the U.S. Department of Defense, and Gateway Institute for Brain Research, Inc. He has received consulting fees from Guidepoint Inc. R.N.A. research is funded by MJFF, the Silverstein Foundation, the Parkinson's Foundation, and the Aufzien Family Center for the Prevention and Treatment of Parkinson's Disease. He received consultation fees from Bexxion, Biogen, Biohaven, Capsida, Gain Therapeutics, Genzyme/Sanofi, Janssen, SK Biopharmaceuticals, Takeda, and Vanqua Bio. D.W. has received research funding or support from MJFF, International Parkinson and Movement Disorder Society (IPMDS), NIH, and the U.S. Department of Veterans Affairs; honoraria for consultancy from AbbVie, Boehringer Ingelheim, CHDI Foundation, Citrus Health Group, Negev Labs, Otsuka, Parkinson Study Group, Sage Therapeutics, Signant Health, and Vanqua Bio; and license fee payments from the University of Pennsylvania for the QUIP, QUIP‐RS, and PDAQ. K.M.: Consultant for MJFF, CPP, Roche, Biohaven, Neuron23, Prothena, Lilly, Calico, ABli, Mitro, BMS, Novartis, and Teva. T.S. declares consultancies for AcureX, Adamas, AskBio, Amneal, Blue Rock Therapeutics, Critical Path for Parkinson's Consortium, Denali, MJFF, Neuroderm, Roche, Sanofi, Sinopia, Takeda, and Vanqua Bio; on advisory boards for AcureX, Adamas, AskBio, Biohaven, Denali, GAIN, Neuron23, and Roche; on scientific advisory boards for Koneksa, Neuroderm, Sanofi, and UCB; and received research funding from Amneal, Biogen, Roche, Neuroderm, Sanofi, Prevail, and UCB; and an investigator for NINDS, MJFF, and Parkinson's Foundation. P.G.L declares an early investigator award from MJFF. N.P. has received consultancy honoraria from Hoffmann‐La Roche, Britannia, Bial, AbbVie, and Teva; speaker fees from Bial, Britannia, AbbVie, GE Healthcare, Boston Scientific; Research Funding from Independent Research Fund Denmark, Danish Parkinson's Disease Association, Parkinson's UK, Center of Excellence in Neurodegeneration (CoEN) network award, GE Healthcare Grant, Multiple System Atrophy Trust, Weston Brain Institute, EU Joint Program Neurodegenerative Disease Research (JPND), EU Horizon 2020 research, MJFF, F. Hoffmann‐La Roche, Medtronic, and Symbyx. K.L.P. is funded by grants from the NIH, MJFF, the Knight Initiative for Brain Resilience, the Wu Tsai Neuroscience Institute, Lewy Body Dementia Association, Parkinson's Foundation, American Parkinson's Disease Association, and the Sue Berghoff LBD Research Fellowship. She has been on the Scientific Advisory Board for Amprion and has been a consultant for Novartis, Lilly, BioArctic, Biohaven, Curasen, and Neuron23.

## Supporting information


**Table S1.** Summary of baseline cerebrospinal fluid alpha‐synuclein seed amplification assay (CSFasynSAA) results in participants with a negative seed amplification assay (SAA−) result on either assay.
**Table S2.** Selected demographic and baseline characteristics of seed amplification assay negative (SAA−) and positive (SAA+) sporadic Parkinson's disease (sPD) participants.
**Table S3.** Extended demographic and baseline characteristics of matched seed amplification assay negative (SAA−) and positive (SAA+) sporadic Parkinson's disease (sPD) participants.
**Table S4.** Baseline magnetic resonance imaging structural analysis from matched seed amplification assay negative (SAA−) and positive (SAA+) sporadic Parkinson's disease (sPD) participants.
**Table S5.** Baseline magnetic resonance imaging structural analysis from unmatched seed amplification assay negative (SAA−) and positive (SAA+) sporadic Parkinson's disease (sPD) participants.
**Table S6.** Seed amplification assay negative (SAA−) sporadic Parkinson's disease (sPD) participant status at year 2.
**Table S7.** Walking and balance milestones met by matched seed amplification assay negative (SAA−) and positive (SAA+) sporadic Parkinson's disease (sPD) participants at first event within 2 years.

## Data Availability

The data that support the findings of this study are openly available in PPMI database at http://www.ppmi-info.org/access-data-specimens/download-data, reference number RRID:SCR_006431.

## References

[1] Postuma RB , Berg D , Stern M , et al. MDS clinical diagnostic criteria for Parkinson's disease. Mov Disord 2015;30(12):1591–1601. 26474316 10.1002/mds.26424

[2] Bega D , Kuo PH , Chalkidou A , et al. Clinical utility of DaTscan in patients with suspected parkinsonian syndrome: a systematic review and meta‐analysis. NPJ Parkinsons Dis 2021;7(1):43. 34031400 10.1038/s41531-021-00185-8 PMC8144619

[3] Quintas S , Sanles‐Falagan R , Berbis MA . I(123)‐FP‐CIT (DaTSCAN) SPECT beyond the most common causes of parkinsonism: a systematic review. Mov Disord Clin Pract 2024;11(6):613–625. 38693679 10.1002/mdc3.14055 PMC11145110

[4] Simuni T , Chahine LM , Poston K , et al. A biological definition of neuronal alpha‐synuclein disease: towards an integrated staging system for research. Lancet Neurol 2024;23(2):178–190. 38267190 10.1016/S1474-4422(23)00405-2

[5] Concha‐Marambio L , Pritzkow S , Shahnawaz M , Farris CM , Soto C . Seed amplification assay for the detection of pathologic alpha‐synuclein aggregates in cerebrospinal fluid. Nat Protoc 2023;18(4):1179–1196. 36653527 10.1038/s41596-022-00787-3 PMC10561622

[6] Rossi M , Candelise N , Baiardi S , et al. Ultrasensitive RT‐QuIC assay with high sensitivity and specificity for Lewy body‐associated synucleinopathies. Acta Neuropathol 2020;140(1):49–62. 32342188 10.1007/s00401-020-02160-8 PMC7299922

[7] Berg D , Klein C . Alpha‐synuclein seed amplification and its uses in Parkinson's disease. Lancet Neurol 2023;22(5):369–371. 37059498 10.1016/S1474-4422(23)00124-2

[8] Grossauer A , Hemicker G , Krismer F , et al. Alpha‐synuclein seed amplification assays in the diagnosis of synucleinopathies using cerebrospinal fluid‐a systematic review and meta‐analysis. Mov Disord Clin Pract 2023;10(5):737–747. 37205253 10.1002/mdc3.13710 PMC10187020

[9] Siderowf A , Concha‐Marambio L , Lafontant DE , et al. Assessment of heterogeneity among participants in the Parkinson's progression markers initiative cohort using alpha‐synuclein seed amplification: a cross‐sectional study. Lancet Neurol 2023;22(5):407–417. 37059509 10.1016/S1474-4422(23)00109-6 PMC10627170

[10] Orru CD , Vaughan DP , Vijiaratnam N , et al. Diagnostic and prognostic value of alpha‐synuclein seed amplification assay kinetic measures in Parkinson's disease: a longitudinal cohort study. Lancet Neurol 2025;24(7):580–590. 40541208 10.1016/S1474-4422(25)00157-7

[11] Kalia LV , Lang AE , Hazrati LN , et al. Clinical correlations with Lewy body pathology in LRRK2‐related Parkinson disease. JAMA Neurol 2015;72(1):100–105. 25401511 10.1001/jamaneurol.2014.2704 PMC4399368

[12] Zimprich A , Biskup S , Leitner P , et al. Mutations in LRRK2 cause autosomal‐dominant parkinsonism with pleomorphic pathology. Neuron 2004;44(4):601–607. 15541309 10.1016/j.neuron.2004.11.005

[13] Marek K , Chowdhury S , Siderowf A , et al. The Parkinson's progression markers initiative (PPMI)–establishing a PD biomarker cohort. Ann Clin Transl Neurol 2018;5(12):1460–1477. 30564614 10.1002/acn3.644 PMC6292383

[14] Ma Y , Farris CM , Weber S , et al. Sensitivity and specificity of a seed amplification assay for diagnosis of multiple system atrophy: a multicentre cohort study. Lancet Neurol 2024;23(12):1225–1237. 39577923 10.1016/S1474-4422(24)00395-8 PMC12288831

[15] Kaasinen V . Ipsilateral deficits of dopaminergic neurotransmission in Parkinson's disease. Ann Clin Transl Neurol 2016;3(1):21–26. 26783547 10.1002/acn3.268 PMC4704477

[16] Fereshtehnejad SM , Moqadam R , Azizi H , et al. Distinct longitudinal clinical‐neuroanatomical trajectories in Parkinson's disease clinical subtypes: insight toward precision medicine. Mov Disord 2025;40(8):1572–1583. 40415649 10.1002/mds.30229 PMC12371632

[17] Zeighami Y , Fereshtehnejad SM , Dadar M , Collins DL , Postuma RB , Dagher A . Assessment of a prognostic MRI biomarker in early de novo Parkinson's disease. Neuroimage Clin 2019;24:101986. 31514113 10.1016/j.nicl.2019.101986 PMC6742805

[18] Zeighami Y , Ulla M , Iturria‐Medina Y , et al. Network structure of brain atrophy in de novo Parkinson's disease. eLife 2015;4:4. 10.7554/eLife.08440 PMC4596689 26344547

[19] Coupe P , Yger P , Prima S , Hellier P , Kervrann C , Barillot C . An optimized blockwise nonlocal means denoising filter for 3‐D magnetic resonance images. IEEE Trans Med Imaging 2008;27(4):425–441. 18390341 10.1109/TMI.2007.906087 PMC2881565

[20] Sled JG , Zijdenbos AP , Evans AC . A nonparametric method for automatic correction of intensity nonuniformity in MRI data. IEEE Trans Med Imaging 1998;17(1):87–97. 9617910 10.1109/42.668698

[21] Xiao Y , Lau JC , Anderson T , et al. An accurate registration of the BigBrain dataset with the MNI PD25 and ICBM152 atlases. Sci Data 2019;6(1):210. 31624250 10.1038/s41597-019-0217-0 PMC6797784

[22] Brumm MC , Siderowf A , Simuni T , et al. Parkinson's progression markers initiative: a milestone‐based strategy to monitor Parkinson's disease progression. J Parkinsons Dis 2023;13(6):899–916. 37458046 10.3233/JPD-223433 PMC10578214

[23] Bukhari SA , Nudelman KNH , Rumbaugh M , et al. Parkinson's Progression Markers Initiative brain autopsy program. Parkinsonism Relat Disord 2022;101:62–65. 35803091 10.1016/j.parkreldis.2022.06.017

[24] Marek K , Russell DS , Concha‐Marambio L , et al. Evidence for alpha‐synuclein aggregation in older individuals with hyposmia: a cross‐sectional study. EBioMedicine 2025;112:105567. 39893720 10.1016/j.ebiom.2025.105567 PMC11835612

[25] Keshtkarjahromi M , Abraham DS , Gruber‐Baldini AL , et al. Confirming Parkinson disease diagnosis: patterns of diagnostic changes by movement disorder specialists. Parkinsons Dis 2022;2022:5535826. 35585902 10.1155/2022/5535826 PMC9110256

[26] Massa J , Chahine LM . Revision of diagnosis in early parkinsonism with abnormal dopamine transporter imaging. J Parkinsons Dis 2019;9(2):327–334. 30958313 10.3233/JPD-181517

[27] Raty V , Kuusimaki T , Majuri J , et al. Stability and accuracy of a diagnosis of Parkinson disease over 10 years. Neurology 2025;104(9):e213499. 40184591 10.1212/WNL.0000000000213499 PMC11970931

[28] Quadalti C , Calandra‐Buonaura G , Baiardi S , et al. Neurofilament light chain and alpha‐synuclein RT‐QuIC as differential diagnostic biomarkers in parkinsonisms and related syndromes. NPJ Parkinsons Dis 2021;7(1):93. 34635674 10.1038/s41531-021-00232-4 PMC8505434

[29] Compta Y , Painous C , Soto M , et al. Combined CSF alpha‐SYN RT‐QuIC, CSF NFL and midbrain‐pons planimetry in degenerative parkinsonisms: from bedside to bench, and back again. Parkinsonism Relat Disord 2022;99:33–41. 35594661 10.1016/j.parkreldis.2022.05.006

[30] Martinez‐Valbuena I , Tartaglia MC , Fox SH , Lang AE , Kovacs GG . Four‐repeat tau seeding in the skin of patients with progressive supranuclear palsy. JAMA Neurol 2024;81(11):1228–1230. 39312261 10.1001/jamaneurol.2024.3162 PMC11420818

[31] Saijo E , Metrick MA 2nd , Koga S , et al. 4‐Repeat tau seeds and templating subtypes as brain and CSF biomarkers of frontotemporal lobar degeneration. Acta Neuropathol 2020;139(1):63–77. 31616982 10.1007/s00401-019-02080-2 PMC7192393

[32] Savica R , Grossardt BR , Bower JH , Ahlskog JE , Rocca WA . Incidence and pathology of synucleinopathies and tauopathies related to parkinsonism. JAMA Neurol 2013;70(7):859–866. 23689920 10.1001/jamaneurol.2013.114 PMC3707980

[33] Robinson JL , Lee EB , Xie SX , et al. Neurodegenerative disease concomitant proteinopathies are prevalent, age‐related and APOE4‐associated. Brain 2018;141(7):2181–2193. 29878075 10.1093/brain/awy146 PMC6022546

[34] Farrer M , Chan P , Chen R , et al. Lewy bodies and parkinsonism in families with parkin mutations. Ann Neurol 2001;50(3):293–300. 11558785 10.1002/ana.1132

[35] Kluge A , Borsche M , Streubel‐Gallasch L , et al. Alpha‐synuclein pathology in PRKN‐linked Parkinson's disease: new insights from a blood‐based seed amplification assay. Ann Neurol 2024;95(6):1173–1177. 38546204 10.1002/ana.26917

[36] Cook L , Verbrugge J , Schwantes‐An TH , et al. Parkinson's disease variant detection and disclosure: PD GENEration, a North American study. Brain 2024;147(8):2668–2679. 39074992 10.1093/brain/awae142 PMC11292896

