# Supplementary material for: Clinical and Imaging Characteristics of Parkinson's Disease with Negative Alpha‐Synuclein Seed Amplification Assay
Source: Mov Disord. 2026 Jan 28;41(5):1114–27. doi: 10.1002/mds.70197 (PMC13206160; doi:10.1002/mds.70197)
Supplement: Supplementary file 1 — Table S1. Summary of baseline cerebrospinal fluid alpha‐synuclein seed amplification assay (CSFasynSAA) results in participants with a negative seed amplification assay (SAA−) result on either assay. Table S2. Selected demographic and baseline characteristics of seed amplification assay negative (SAA−) and positive (SAA+) sporadic Parkinson's disease (sPD) participants. Table S3. Extended demographic and baseline characteristics of matched seed amplification assay negative (SAA−) and positive (SAA+) sporadic Parkinson's disease (sPD) participants. Table S4. Baseline magnetic resonance imaging structural analysis from matched seed amplification assay negative (SAA−) and positive (SAA+) sporadic Parkinson's disease (sPD) participants. Table S5. Baseline magnetic resonance imaging structural analysis from unmatched seed amplification assay negative (SAA−) and positive (SAA+) sporadic Parkinson's disease (sPD) participants. Table S6. Seed amplification assay negative (SAA−) sporadic Parkinson's disease (sPD) participant status at year 2. Table S7. Walking and balance milestones met by matched seed amplification assay negative (SAA−) and positive (SAA+) sporadic Parkinson's disease (sPD) participants at first event within 2 years. [file MDS-41-1114-s001.docx]

## **Supplementary Table 1. Summary of baseline CSFasynSAA results in participants with SAA- result on either assay.**

| **Result Pattern (150h Baseline ∕ 24h Baseline)** | **N** |
| --- | --- |
| **Included in SAA- cohort** | |
| 0 / 0 | 13 |
| 0 / X | 9* |
| 3 / 0 | 1 |
| X / 0 | 57 |
| **Excluded from SAA- cohort (due to prioritization of 24h assay)** | |
| 0 / 1 | 3 |
| 0 / 2 | 3 |

0 = Negative, 1 = Positive, 2 = MSA-like, 3 = inconclusive, X = no result available.

* Of the 9 participants for whom only the 150hr CSFasynSAA was available at baseline, 7 had the 24hr assay performed at a follow-up visit and the follow-up 24hr result was negative in all 7 cases.

## **Supplementary Table 2. Selected demographic and baseline characteristics of SAA- and SAA+ sporadic PD participants.**

| **Variable** | **SAA- sPD (N = 80)** | **SAA+ sPD (N = 856)** | ***p*-value^a^** |
| --- | --- | --- | --- |
| **Age at enrollment, years**, median (IQR) | 66.8 (61.7–73.1) | 63.9 (57.2–69.9) | 0.001 |
| Mean (SD) | 66.6 (9.1) | 63.2 (9.4) |  |
| **Male sex**, n (%) | 51 (64%) | 560 (65%) | 0.764 |
| **Time since diagnosis at enrollment**, median (IQR) | 0.5 (0.3–0.8) | 0.5 (0.3–1.0) | 0.604 |
| Mean (SD) | 0.6 (0.5) | 0.7 (0.6) |  |
| Missing | 1 | 0 |  |

^a^Comparisons by SAA status used Chi-Square or Fisher's Exact tests for categorical variables and Wilcoxon rank sum tests for continuous variables.

## **Supplementary Table 3. Extended demographic and baseline characteristics of matched SAA- and SAA+ sporadic PD participants.**

| **Variable** | **SAA- sPD (N = 79^a^)** | **SAA+ sPD (N = 237)** | ***p*-value^b^** |
| --- | --- | --- | --- |
| **Age at enrollment, years**, median (IQR) | 66.7 (61.3–73.3) | 67.4 (62.1–72.5) | 0.959 |
| Mean (SD) | 66.5 (9.2) | 66.6 (8.6) |  |
| **Male sex**, n (%) | 50 (63%) | 150 (63%) | 1.000 |
| **Education > 12 years**, n (%) | 65 (82%) | 206 (87%) | 0.266 |
| Missing | 0 | 1 |  |
| **Family history of PD**, n (%) |  |  | 0.752 |
| 1st-degree family w/ PD | 10 (13%) | 37 (16%) |  |
| Non-1st-degree family w/ PD | 9 (11%) | 30 (13%) |  |
| No family w/ PD | 60 (76%) | 170 (72%) |  |
| **Time since diagnosis at enrollment**, median (IQR) | 0.5 (0.3–0.8) | 0.5 (0.3–0.8) | 0.369 |
| Mean (SD) | 0.6 (0.5) | 0.6 (0.5) |  |
| **Duration of follow-up from BL, years**, median (IQR) | 1.9 (1.0–3.0) | 2.1 (1.0–6.0) | 0.060 |
| **Hoehn & Yahr stage**, n (%) |  |  | 0.201^c^ |
| 1 | 19 (24%) | 75 (32%) |  |
| 2 | 59 (75%) | 161 (68%) |  |
| 3 | 1 (1%) | 1 (<1%) |  |
| **Race**, n (%) |  |  | 0.318^c^ |
| White | 75 (95%) | 215 (91%) |  |
| Black or African American | 4 (5%) | 6 (3%) |  |
| Asian | 0 | 4 (2%) |  |
| Other | 0 | 10 (4%) |  |
| Missing | 0 | 2 |  |
| **Hispanic or Latino ethnicity**, n (%) | 4 (5%) | 6 (3%) | 0.278 |
| Missing | 0 | 2 |  |
| **UPSIT percentile**, median (IQR) | 55.0 (26.0–78.5) | 8.0 (4.0–16.0) | <.001 |
| Missing | 1 | 4 |  |
| **UPSIT ≤ 15th %ile**, n (%) | 9 (12%) | 171 (73%) | <.001 |
| Missing | 1 | 4 |  |
| **MDS-UPDRS Part I**, median (IQR) | 6.0 (4.0–11.0) | 5.0 (3.0–8.0) | 0.069 |
| Missing | 1 | 2 |  |
| **MDS-UPDRS Part II**, median (IQR) | 7.0 (4.0–11.0) | 5.0 (3.0–8.0) | 0.003 |
| Missing | 0 | 1 |  |
| **MDS-UPDRS Part III (OFF)**, median (IQR) | 22.5 (18.0–27.0) | 22.0 (15.5–29.0) | 0.648 |
| Missing | 1 | 1 |  |
| **Motor symptom asymmetry index (OFF)**, median (IQR) | 0.40 (0.20–0.60) | 0.52 (0.32–0.81) | 0.001 |
| Missing | 0 | 1 |  |
| **Tremor score (OFF)**, median (IQR) | 4.0 (2.0–7.0) | 5.0 (3.0–7.0) | 0.075 |
| **Postural tremor subscore (OFF)**, median (IQR) | 1.0 (0.0–2.0) | 1.0 (0.0–1.0) | 0.324 |
| **Kinetic tremor subscore (OFF)**, median (IQR) | 1.0 (0.0–2.0) | 1.0 (0.0–2.0) | 0.671 |
| **Rest tremor subscore (OFF)**, median (IQR) | 2.0 (0.0–4.0) | 4.0 (2.0–5.0) | 0.002 |
| **MDS-UPDRS Total Score (OFF)**, median (IQR) | 39.0 (28.0–48.0) | 32.0 (25.0–43.0) | 0.020 |
| Missing | 2 | 3 |  |
| **Modified Schwab & England**, median (IQR) | 95.0 (90.0–100.0) | 95.0 (90.0–100.0) | 0.245 |
| **MoCA**, median (IQR) | 27.0 (25.0–29.0) | 27.0 (25.0–29.0) | 0.531 |
| Missing | 0 | 2 |  |
| **Cognitive categorization**, n (%) |  |  | 0.244 |
| Normal | 54 (83%) | 150 (89%) |  |
| Mild cognitive impairment | 11 (17%) | 19 (11%) |  |
| Missing | 14 | 68 |  |
| **RBDSQ**, median (IQR) | 3.0 (2.0–6.0) | 3.0 (2.0–5.0) | 0.912 |
| Missing | 0 | 4 |  |
| **pRBD (RBDSQ ≥ 6)**, n (%) | 20 (25%) | 51 (22%) | 0.530 |
| Missing | 0 | 4 |  |
| **GDS**, median (IQR) | 2.0 (1.0–5.0) | 2.0 (0.0–3.0) | 0.025 |
| **SCOPA-AUT**, median (IQR) | 11.0 (7.0–14.0) | 9.0 (6.0–13.0) | 0.151 |
| Missing | 2 | 3 |  |
| **SCOPA-AUT Gastrointestinal Score**, median (IQR) | 2.0 (1.0–4.0) | 2.0 (1.0–4.0) | 0.827 |
| Missing | 1 | 2 |  |
| **SCOPA-AUT Urinary Score**, median (IQR) | 5.0 (3.0–6.0) | 4.0 (3.0–6.0) | 0.534 |
| Missing | 1 | 2 |  |
| **SCOPA-AUT Cardiovascular Score**, median (IQR) | 0.0 (0.0–1.0) | 0.0 (0.0–1.0) | 0.277 |
| Missing | 1 | 1 |  |
| **SCOPA-AUT Thermoregulatory Score**, median (IQR) | 1.0 (0.0–2.0) | 1.0 (0.0–2.0) | 0.209 |
| Missing | 1 | 1 |  |
| **SCOPA-AUT Pupillomotor Score**, median (IQR) | 0.0 (0.0–1.0) | 0.0 (0.0–1.0) | 0.700 |
| Missing | 1 | 1 |  |
| **SCOPA-AUT Sexual Score**, median (IQR) | 0.0 (0.0–2.0) | 0.0 (0.0–2.0) | 0.581 |
| Missing | 2 | 1 |  |
| **Lowest putamen ratio**, median (IQR) | 0.35 (0.23–0.60) | 0.35 (0.29–0.43) | 0.736 |
| Missing | 4 | 2 |  |
| **Mean striatum binding**, median (IQR) | 1.29 (1.03–1.83) | 1.40 (1.18–1.67) | 0.557 |
| Missing | 4 | 2 |  |
| **Mean caudate binding**, median (IQR) | 1.87 (1.45–2.33) | 1.93 (1.66–2.35) | 0.188 |
| Missing | 4 | 2 |  |
| **Mean putamen binding**, median (IQR) | 0.84 (0.56–1.39) | 0.84 (0.68–1.05) | 0.704 |
| Missing | 4 | 2 |  |
| **DAT binding asymmetry index**, median (IQR) | 0.12 (0.07–0.20) | 0.15 (0.08–0.25) | 0.169 |
| Missing | 4 | 2 |  |

UPSIT = University of Pennsylvania Smell Identification Test; MDS-UPDRS = Movement Disorder Society-Unified Parkinson's Disease Rating Scale; MoCA = Montreal Cognitive Assessment; RBDSQ = REM Sleep Behavior Disorder-Screening Questionnaire; GDS = Geriatric Depression Scale; SCOPA-AUT = Scales for Outcomes in Parkinson's Disease-Autonomic

^a^One SAA- sPD participant excluded from matched analysis due to missing disease duration.

^b^Comparisons by SAA status used Chi-Square or Fisher's Exact tests for categorical variables and Wilcoxon rank sum tests for continuous variables.

^c^For the purposes of comparisons, Hoehn & Yahr stage was dichotomized as stage 1 vs. ≥ 2, and race was dichotomized as White vs. other.

##

## **Supplementary Table 4. Baseline MRI structural analysis from matched SAA- and SAA+ sPD participants**

| Region | Estimate | T-value | P-value |
| --- | --- | --- | --- |
| Left red nucleus | -0.7 | -3.44 | 0.000817497868509641 |
| Left substantia nigra | -0.64 | -3.03 | 0.00298901796475307 |
| Right substantia nigra | -0.7 | -3.26 | 0.00148094605841368 |
| Left subthalamic nucleus | -0.83 | -4.23 | 4.66304788499494E-05 |
| Right subthalamic nucleus | -0.72 | -3.81 | 0.000217608155873354 |
| Left globus pallidus externa | -0.83 | -4.36 | 2.72791419492869E-05 |
| Right globus pallidus externa | -0.63 | -3.07 | 0.00265822104432756 |
| Left globus pallidus interna | -0.98 | -5.1 | 1.28699784513741E-06 |
| Right globus pallidus Interna | -0.66 | -3.2 | 0.00175021191360466 |

## **Supplementary Table 5. Baseline MRI structural analysis from unmatched SAA- and SAA+ sPD participants**

| Region | Estimate | T-value | P-value |
| --- | --- | --- | --- |
| Left red nucleus | -0.53 | -3.07 | 0.00233992397240574 |
| Left substantia nigra | -0.54 | -2.83 | 0.00491841789504269 |
| Right substantia nigra | -0.58 | -3.02 | 0.0027473274785897 |
| Left subthalamic nucleus | -0.86 | -4.91 | 1.30793162711068E-06 |
| Right subthalamic nucleus | -0.73 | -4.28 | 2.39023871929471E-05 |
| Left putamem | -0.56 | -3.09 | 0.00215668355670961 |
| Left globus pallidus externa | -0.99 | -5.41 | 1.06916053191345E-07 |
| Right globus pallidus externa | -0.73 | -3.94 | 9.88996669618816E-05 |
| Left globus pallidus interna | -1.19 | -6.47 | 2.76862101826289E-10 |
| Right globus pallidus Interna | -0.77 | -4.16 | 3.94192207551866E-05 |

## **Supplementary Table 6. SAA- sporadic PD participant status at year 2.**

| **Status (at year 2)** | **All Participants (n = 80)** | **Pre-2020 Participants (n = 22)** | **Post-2020 Participants (n = 58)** |
| --- | --- | --- | --- |
| Completed^a^ | 42 (53%) | 20 (91%) | 22 (38%) |
| Not yet due | 31 (39%) | 0 | 31 (53%) |
| Lost to follow-up (1.0)^b^ *or* overdue (2.0) | 3 (4%) | 1 (5%) | 2 (3%) |
| Withdrew before reaching visit | 4 (5%) | 1 (5%) | 3 (5%) |

^a^19 participants withdrew/completed study after completing the following annual visit: year 2 (n = 4), year 3 (n = 5), year 4 (n = 2), year 5 (n = 2), year 6 (n = 1), year 7 (n = 2), year 9 (n = 1), year 10 (n = 2).

^b^1 participant did not complete year 2 visit and withdrew before completing subsequent visit.

##

## **Supplementary Table 7. Walking and balance milestones met by matched SAA- and SAA+ sporadic PD participants at first event within 2 years.**

| **Variable** | **SAA- sPD (N = 67)** | **SAA+ sPD (N = 209)** |
| --- | --- | --- |
| **Any walking and balance milestone** | **9 (13%)** | **6 (3%)** |
| Postural instability (item 3.12 ≥ 3 [ON or OFF]) | 6 (9%) | 3 (1%) |
| Walking and balance (item 2.12 ≥ 3) | 4 (6%) | 2 (1%) |
| Hoehn & Yahr (≥ 4 [ON or OFF]) | 2 (3%) | 1 (<1%) |
| Gait (item 3.10 ≥ 3 [ON or OFF]) | 2 (3%) | 2 (1%) |
| Freezing (item 2.13 ≥ 3) | 2 (3%) | 0 |
| Freezing of gait (item 3.11 = 4 [ON or OFF]) | 0 | 0 |

MDS-UPDRS = Movement Disorder Society-Unified Parkinson's Disease Rating Scale

Data only considers the *initial* event (i.e., first visit at which criteria for at least one walking and balance milestone were met). Columns include participants who did not meet any of the survival outcomes at baseline (i.e., excludes 12 SAA- and 28 SAA+ participants who met any of the survival outcomes at baseline, of whom 3 and 2 participants met a walking and balance milestone, respectively).

##

## **Supplementary Figure 1. Time to progression milestones in matched SAA- and SAA+ sporadic PD participants excluding SAA- participants who had change in primary research diagnosis**


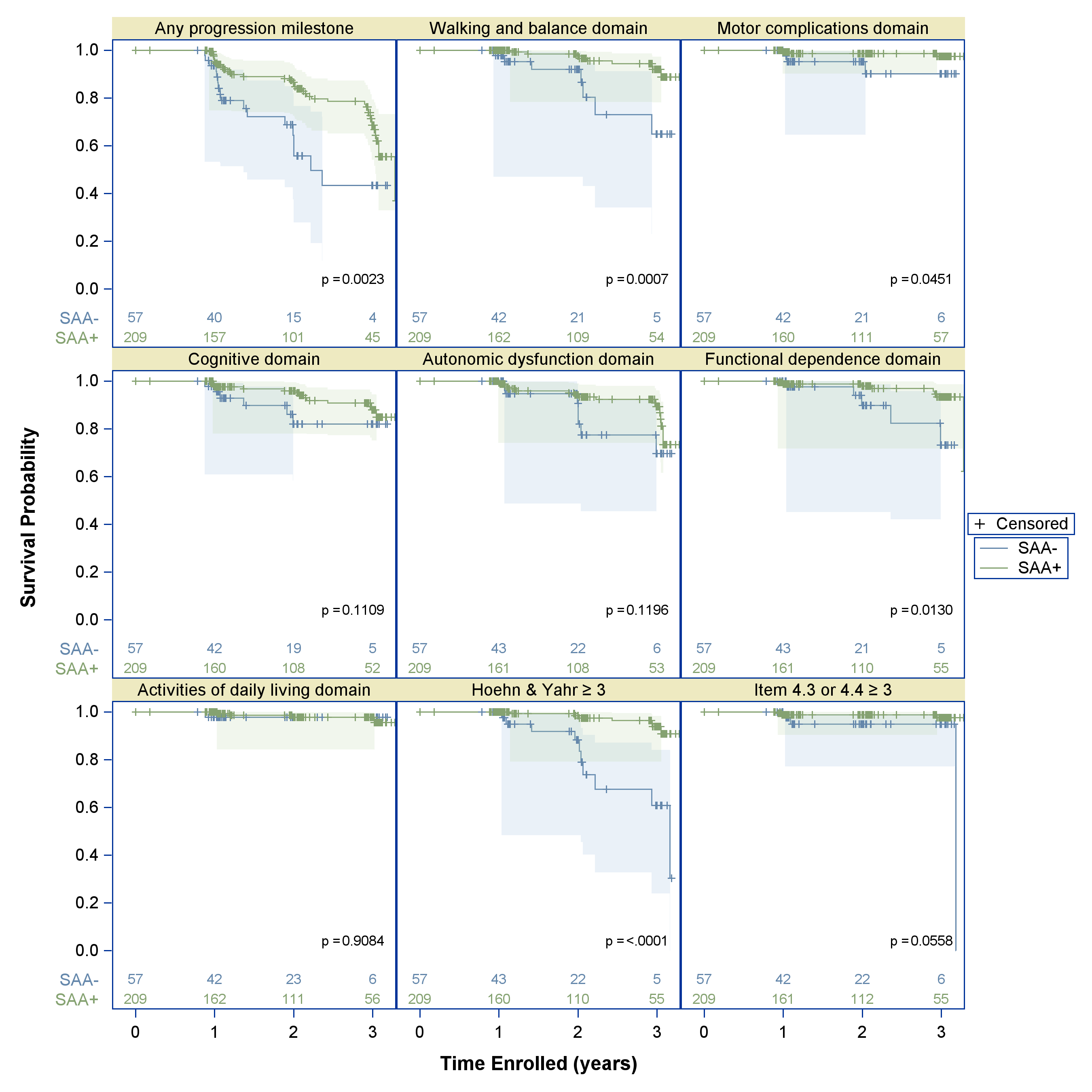


## 
